# Leveraging on large language model to classify sentences: a case study applying STAGES scoring methodology for sentence completion test on ego development

**DOI:** 10.3389/fpsyg.2025.1488102

**Published:** 2025-02-06

**Authors:** Xavier Bronlet

**Affiliations:** Integral Transpersonal Institute, Milan, Italy

**Keywords:** large language model, automated classification, ego development, Cohen’s kappa, methods

## Abstract

**Introduction:**

The emergence of artificial intelligence and the widespread availability of large language model open the door to text analysis at scale leveraging on complex classification instructions. This case study explores the possibility of using available large language models to measure ego development at scale and establish a methodology that can be applied to other classification instructions. Ego consists of the traits that influence how a person perceives and engages with the world, while ego development is a crucial aspect of adult personality growth, influencing behaviors and decisions in both personal and professional contexts. Accurate assessments of ego development stages are vital for creating effective strategies in organizational psychology and corporate analytics.

**Methods:**

This case study investigates the agreement between expert and automated classifications of ego development stages, aiming to evaluate the potential of automation in this domain leveraging artificial intelligence and large language models. Cohen’s kappa statistic has been used to measure the agreement between classifications made by experts and those generated by an automated process leveraging large language models.

**Results:**

The comparison between the scoring of experts and large language models yielded a weighted Kappa value of 0.779, indicating a substantial level of agreement that is statistically meaningful and unlikely to be due to chance.

**Discussion:**

While this suggests valuable scoring that leverages large language models, it also highlights the opportunity for further refinement to closely match expert assessments. We observed low variability in aggregated values, demonstrating that the automated process functions effectively at scale. The robustness of aggregated data is particularly evident when calculating ego development scores for individuals, groups, corporate units, and entire corporations. This capability underscores the utility of the automated system for high-level evaluations and decision-making leveraging on a solid indicator. While the classification system developed in this case study shows promise, targeted enhancements may help to achieve a level of accuracy and reliability that improves alignment with experts’ evaluations for single sentences. The methodology developed in this case study appears to be useful to support other evaluations at scale that leverage large language models using other maps of classifications.

## Introduction

In the rapidly evolving landscape of today’s world, characterized by complex social dynamics, technological advancements, and global interconnectedness, the concept of “ego development” assumes paramount importance (O'Fallon et al., 2020). Ego development, as conceptualized by Jane Loevinger, Suzanne Cook-Greuter, and other developmental psychologists, refers to the process through which individuals evolve in their understanding of themselves and their relationships with others, also called meaning-making maturity or complexity, ego development, or perspective-taking capacity. This evolution encompasses a spectrum from simple, self-centered perspectives to more complex, integrated ways of relating to the world (Kroeber, 2006).

In contemporary society, the significance of ego development is multifaceted. As individuals navigate through various stages of life, their ability to effectively manage personal and interpersonal challenges is often contingent upon the maturity of their ego (Fleischer, 2023). Higher levels of ego development are associated with enhanced emotional regulation, greater empathy, improved problem-solving skills, and a more nuanced understanding of societal complexities. These attributes are increasingly valuable in a world where collaboration, cultural sensitivity, and psychological resilience are critical for both personal success and collective wellbeing (Balland et al., 2022).

Moreover, the rapid technological advancements and pervasive digital interactions of the modern era necessitate a robust framework for self-awareness and ethical reasoning—core components of advanced ego development (McLennan et al., 2020). With the omnipresence of social media and digital communication, individuals are constantly exposed to diverse viewpoints and must navigate the pressures of identity representation and relational dynamics in virtual spaces (Aichner et al., 2021). Consequently, the progression of ego development can enable individuals to maintain a grounded sense of self while authentically engaging with a broader, interconnected global community (Scardamalia et al., 2012).

Given its profound impact on personal and societal functioning, there exists a burgeoning interest in the measurement of ego development. Quantifying ego development provides invaluable insights into the psychological growth trajectories of individuals, allowing researchers, clinicians, educators, and policymakers to tailor interventions and support systems more effectively (O'Fallon et al., 2020).

### Clinical applications

In therapeutic settings, understanding a patient’s ego development stage can guide the formulation of treatment plans that align with their cognitive and emotional capacities. Therapists can employ strategies that are congruent with the client’s current level of self-understanding and relational patterns, fostering more significant therapeutic progress (Noam, 1998).

### Educational and developmental interventions

In educational contexts, assessing ego development can inform the design of curricula and developmental programs that promote socio-emotional learning and critical thinking. Educators can facilitate experiences that challenge students to advance through successive stages of ego development, ultimately nurturing well-rounded, psychologically mature individuals (Preszler Weathersby, 1998).

### Organizational and leadership development

In the realm of organizational psychology, measuring ego development can aid in identifying leadership potential and fostering organizational cultures that value growth-oriented mindsets. Leaders with higher levels of ego development are more adept at navigating complex decision-making environments, fostering inclusive work cultures, and leading with integrity (Eigel and Kuhnert, 2005).

### Policy and social programs

Policymakers and social program designers can benefit from understanding the ego development levels prevalent within different populations. Such knowledge can inform the creation of initiatives that address the specific developmental needs of various demographic groups, promoting societal wellbeing and cohesion as spiral dynamics and its application in understanding and addressing complex societal issues, such as apartheid in South Africa (Beck et al., 2018).

In summary, the relevance of ego development in today’s context cannot be overstated. As individuals and societies collectively confront an array of contemporary challenges, fostering advanced stages of ego development emerges as a critical goal. Through the precise measurement and promotion of ego development, we can better equip individuals to thrive personally and contribute meaningfully to the collective fabric of society (O'Fallon et al., 2020).

So far, ego development has been assessed by applying specific protocols driven by experts such as the Subject-Object Interview established by Robert Kegan (Berger and Atkins, 2009) or the STAGES framework authored by O’Fallon and based on a sentence completion test established by Jane Loevinger and adapted by Suzanne Cook-Greuter (O'Fallon et al., 2020). The input provided by the respondents is functional to the establishment of the ego development level by a trained expert who can decipher the clues nidified in the narratives. While a more technical analysis leads to the selection of a label that qualifies the ego development (O'Fallon et al., 2020), a deeper review of content is useful to nurture the analysis and provides the expert with qualitative insights derived from the way the respondent instantiates precise archetype such as children, the mother, and the father (Cook-Greuter, 2011). Such protocols require time and trained experts, their scalability on a larger scale requires large efforts as the protocols are adapted to support vertical development of individuals. What about using such protocols on a collective unit of analysis? What are the ways to port such protocols in groups or corporations to capture the general level of thinking or meaning-making maturity and the peculiarities of each operating unit? In groups, checking distributions and outliers may be useful in a context that promotes diversity and inclusion.

The emergence of a powerful large language model (LLM) and in general artificial intelligence may offer a valid solution to assess the level of thinking in groups and other larger datasets (Abburi et al., 2023). In no way, this case study intends to open the door to automated assessment for individual development. This case study explores the feasibility and potential validity of such an approach. Several scenarios have been explored using local large model languages such as Llamafile (LLaMA-3-Instruct-70B) and the most common LLM accessible via API such as ChatGPT 3.5, ChatGPT 4, and Claude 3. The purpose of the case study is not to offer a comparison of the results obtained with the various methods but to report on the most convincing LLM.

## Literature review

While ego development and in particular the STAGES scoring methodology have been widely discussed in literature, the usage of the large language model for text classification is an emerging subject for which the literature is still enriching on a day-by-day basis. The effervescence around artificial intelligence is growing, and literature follows this trend. The case study has considered the body of knowledge available at the very moment.

### Ego and ego development

The ego consists of the traits that influence how a person perceives and engages with the world. It develops to address external challenges such as fear and tolerance. This capacity to differentiate between what is demanded externally and one’s own behavior fosters the development of more sophisticated cognitive processes, including thinking, imagining, and planning (Loevinger, 1976). The ego is not a static set of traits but a dynamic process that grows more sophisticated over time. Each stage represents a new level of consciousness and understanding (Kegan, 1979).

**Figure 1:**
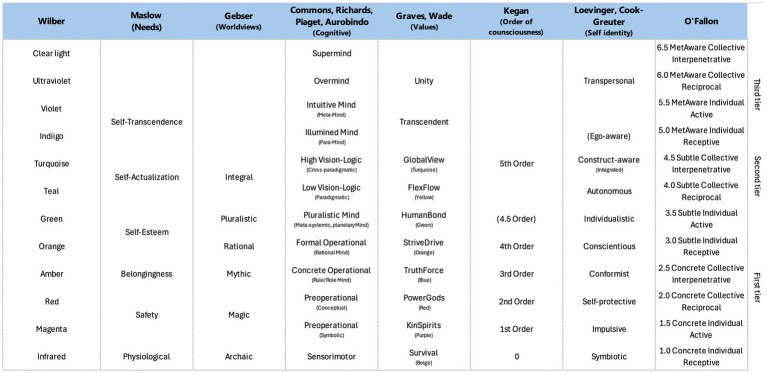
Associated view on consciousness scale from various authors (O'Fallon et al., 2020; Wilber, 2007).

Various field researchers have elaborated their own scale to represent ego development also called level of consciousness. If each of them is looking at the evolution through specific lenses, there is a correspondence between those scales (Figure 1).

### The STAGES level model

A sentence completion test (SCT) can be used to position respondents to the scale suggested by Loevinger and reviewed by Cook-Greuter. This test, first known under the name Washington University Sentence Completion Test (WUSCT), has been elaborated by Loevinger followed by various revisions from which the major one by Cook-Greuter. One of the last versions of the test is made of 32 stems to be completed by the respondent, STAGES scoring protocol authored by O’Fallon results to agree with CG/L protocol authored by Cook-Greuter (O'Fallon et al., 2020).

STAGES builds on research from the highly validated WUSCT. It offers an underlying structural explanation for Cook-Greuter’s system based on three dimensions. Two of these dimensions are polar factors: individual/collective and passive/active. The third dimension categorizes the sophistication of the types of objects referenced, identifying them as Concrete, Subtle/abstract, or “Metaware” (O'Fallon et al., 2020).

STAGES protocol is based on the analysis of the sentences written by the respondent to answer those three questions:

Question 1: What is the category of object awareness: Concrete, Subtle, or MetAware?

Question 2: Does it foreground Individual or Collective objects?

Question 3: Is the cognitive orientation receptive (simple passive), active (simple active), reciprocal (complex with passive predominating), or interpenetrative (complex with active predominating)?

**Figure 2:**
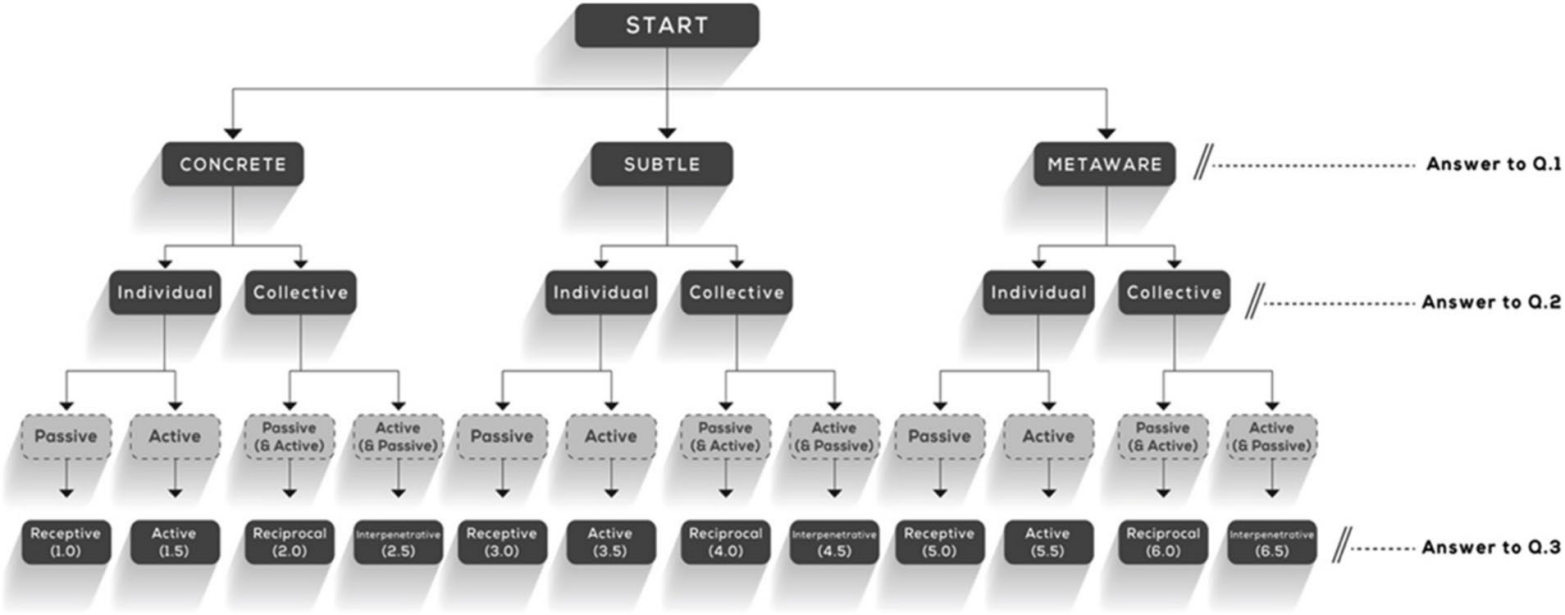
Twelve levels in the STAGES model (O'Fallon et al., 2020).

The classification into one of the 12 possible levels of the STAGES model follows the decision tree described in Figure 2.

### Accuracy of experts scoring

Despite a precise protocol defined to score the sentences, a variability between expert scorers is observed and STAGES experts receive their certification when the goodness of their scoring is above 85% (Merckoll, 2023). The intrinsic nature of the vocabulary used to complete the stems combined with the conjunctions of multiple ideas in a single sentence leads to a potential variability among the experts.

### Text classification using a large language model

Recently, large language models (LLMs) have driven significant advancements in various natural language processing fields. Interest in artificial intelligence among both academics and the public surged with the release of ChatGPT of OpenAI in late 2022. LLMs hold the potential to unlock new opportunities for social scientific research, as these cutting-edge models can be readily adapted to new tasks and demonstrate remarkable performance with relatively minimal training (Wankmüller, 2022). The generative capabilities of these models extend beyond traditional text analysis, opening up new avenues for social scientific research through the creation of machine-generated texts (Argyle et al., 2023).

The LLMs have the capabilities to emulate human-like cognitive processes, including reasoning, creativity, and decision-making. These models are being explored as tools for literature review, hypothesis generation, experimental design, data analysis, and even replacing human participants in certain psychological experiments. Their applications span cognitive and behavioral psychology, clinical and counseling psychology, educational and developmental psychology, and social and cultural psychology. LLMs have been shown to simulate human-like decision-making and emotional responses and aid in mental health assessments and personalized interventions (Guo et al., 2024).

Large language models are the latest breakthrough in supervised text classification. Recent studies highlight the superior performance of these models over traditional machine learning algorithms in a range of sociologically relevant classification tasks, including detecting emotional language (Widmann and Wich, 2022), identifying nationalist, populist, and authoritarian rhetoric (Bonikowski et al., 2022), and classifying offensive language (Wankmüller, 2022).

Large language models offer remarkable possibilities for processing and generating vast amounts of language data. Given the centrality of language in psychology, these technologies hold significant potential to reshape the field. While LLMs show promise for advancing psychological measurement, research, and practice, they are not yet equipped for many transformative applications in psychology. However, with continued research and refinement, their use in such areas may become feasible (Demszky et al., 2023).

The advent of generative AI tools is poised to profoundly influence sentiment analysis research. Large language models (LLMs) often rival or even surpass high-performing transfer learning models in sentiment classification accuracy, making them ideal for direct integration into business applications. Their performance, however, can be significantly affected by factors such as analytical methodologies, linguistic properties, and data characteristics, including text origin and length (Martin and Jurafsky, 2009; Krugmann and Hartmann, 2024).

## Methodology

This case study outlines the development and evaluation of a protocol designed to implement STAGES scoring at scale using large language models (LLMs). The methodology consists of several key steps aimed at optimizing the protocol, validating its accuracy, and ensuring its applicability for large-scale use.

### Protocol development and validation

The protocol was developed using a training set of sentences, while its accuracy and variability were validated using a test set. Both sets were independently rated by expert scorers using a scoring framework distinct from STAGES, with an observed agreement level of 85% between the two methods (Merckoll, 2023). Cohen’s weighted kappa was employed to measure the inter-rater agreement between the automated scoring and expert manual ratings of the protocol.

The protocol was implemented through a Python script that interacts with selected LLMs to classify sentences based on the three questions integral to the STAGES scoring method. Classification prompts were tailored to enable LLMs to provide accurate and consistent responses.

### Steps in the protocol development

1. Training and Test Sentences:
  Training sentences were used to refine prompts and optimize the classification script. These sentences were not used for fine-tuning the pre-trained data of the LLM (Rafailov et al., 2023). Instead, they served to empirically improve the protocol’s scoring capabilities.
  Test sentences, rated by experts using the MAP scoring framework, were employed to evaluate the performance of the protocol at scale (Table 3).

2 LLM Selection (Viera and Garrett, 2005):
  Multiple LLMs, including GPT-3.5 Turbo, GPT-4, GPT-4 Turbo, Claude Sonnet, Claude Opus, and LLaMA 3 70b, were evaluated against the training set. The model demonstrating the highest agreement with expert classifications using the initial prompts was selected for subsequent optimization.

3 Prompt Engineering:
  Prompts were iteratively refined using training data to minimize discrepancies between expert ratings and LLM classifications. Empirical methods were employed to identify patterns in misclassified sentences and integrate these insights into the prompts (Wang et al., 2024).

4 Ensuring Deterministic Scoring:
  To control output variability, the temperature parameter of the LLM was set to zero, ensuring deterministic responses. Additionally, nucleus sampling (top-p) was adjusted to further reduce stochasticity (Chae and Davidson, 2024; Ravfogel et al., 2023). This was critical as the STAGES level relies on consistent answers to three interconnected questions, and variability in one response could alter the final classification (Aichberger et al., 2024).

5 Determining Minimum Sentence Requirements:
  The study emphasized optimizing the number of sentences required for scoring, focusing on deriving an average STAGES level for groups rather than individual sentences. This approach balances precision with the efficiency of respondent participation.

6 Protocol Validation at Scale:
  The efficacy of the protocol was validated using test sentences rated by expert scorers. Cohen’s weighted kappa was calculated to measure agreement between the automated protocol and expert classifications, ensuring reliability for large-scale applications (Vanbelle, 2016).

By integrating LLMs with an optimized protocol, this methodology demonstrates a scalable approach for implementing STAGES scoring. The systematic evaluation and iterative refinement of prompts, combined with robust statistical validation, provide a reliable framework for large-scale language analysis.

## Case study

### Choosing the appropriate LLM

To find the best suitable LLM for the case study, GPT-3.5-turbo, GPT-4o, GPT-4, GPT-4-turbo, Claude Sonnet, Claude Opus, and LLaMA 3 70b have been tested against a sample of 32 sentences, and the results compared with the manual classification performed by an expert. An initial set of prompts has been used, the same for each LLM.

**Figure 3:**
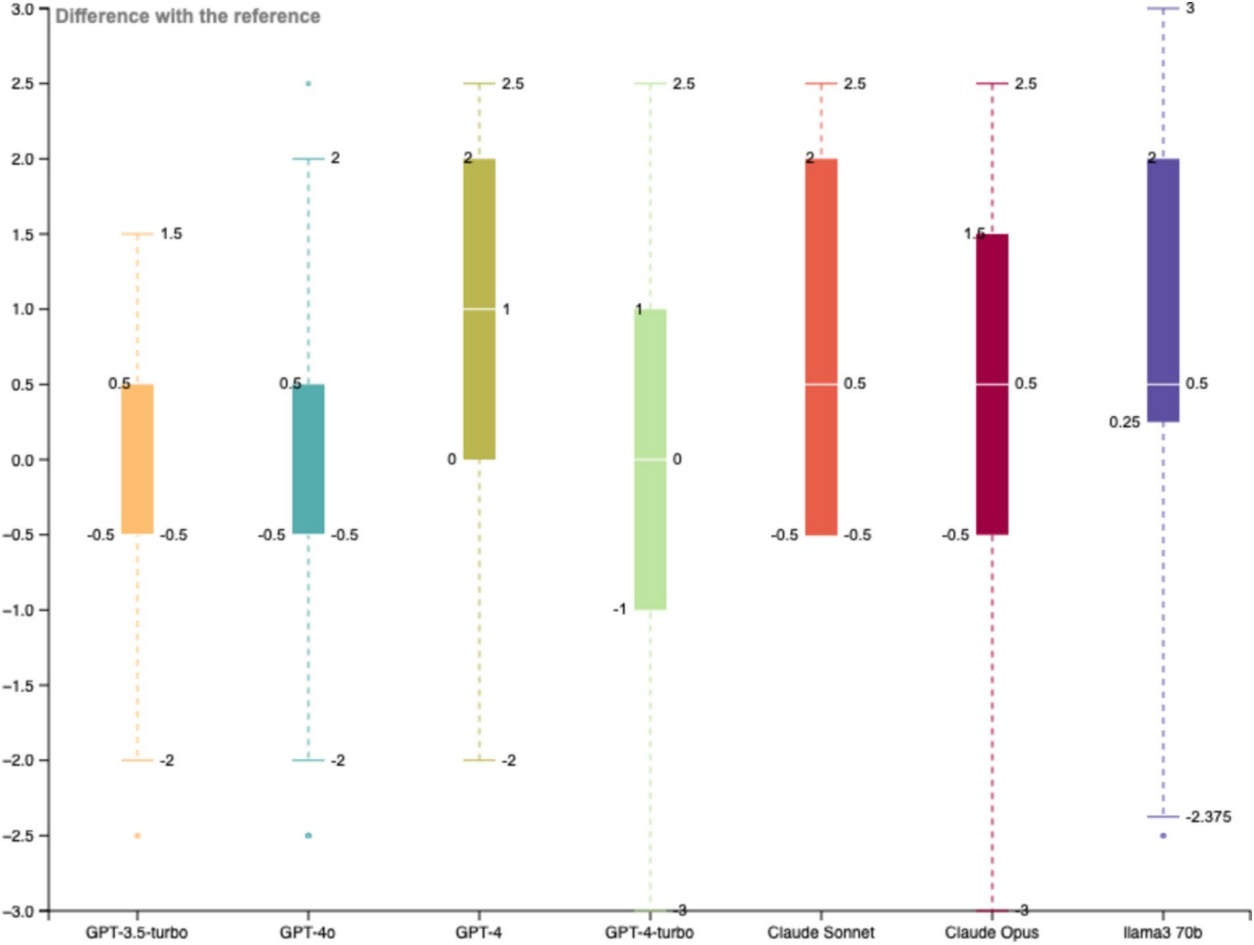
Variability analysis with all LLMs on the training dataset.

A variability analysis has been carried forward comparing the distribution of the differences of each LLM with respect to the expert scoring. The lower variability has been observed with GPT-3.5-turbo for which the standard deviation of the differences between LLM and expert scoring is the lower and the average valuation of the 32 sentences is the nearest to the one established by the expert (average scoring of 3.906 for GPT-3.5-turbo and average scoring of 3.969 for the expert), the second best model is GPT-4o (average scoring of 3.844). Considering the larger context window of 128′000 tokens with a maximum output token of 16′384 tokens available for the model GPT-4o, this model is preferred to exchange a larger amount of data during the classification process (Figure 3 and Table 1).

**Table 1:** Variability analysis of the LLMs compared to expert scoring.

LLM	Min difference	Max difference	Std. dev.	Average valuation
GPT-3.5-turbo	0	2.5	0.634	3.906
GPT-4	0	2.5	0.942	3.547
GPT-4-turbo	0	3	0.924	4.906
GPT-4o	0	2.5	0.884	3.844
Claude Opus	0	3	0.982	4.141
Claude Sonnet	0	2.5	0.860	4.859
LLaMA 3 70b	0.5	3	0.869	4.484
Expert	0	0	0	3.969

### Prompt engineering

The initial prompt to the LLM defined for each of the three questions to be answered is reviewed to reduce the variability over multiple runs and accuracy to reproduce expert scoring results. A thorough analysis of the sentences not perfectly classified helps improve prompt instructions. It is observed that in certain cases, too many instructions lead to confusing results calling for a simplification of the prompt. The final prompts to answer the three questions are as follows:

Is the sentence Concrete, Subtle, or Metaware?

*Classify the following sentence as (1) Concrete, (2) Subtle, or (3) MetAware according to Terri O’Fallon’s classification protocol.*

*A Concrete sentence refers to tangible objects or phenomena directly perceivable through the senses or personal experiences tied to specific, observable events. It often involves physical objects, direct sensory descriptions, or basic actions. Examples include “The car is red,” “I heard a melody,” and “She followed the rules.”*

*A Subtle sentence refers to abstract concepts, ideas, or phenomena not directly tied to sensory experience. It often involves reflection, moral or ethical considerations, theoretical reasoning, future possibilities, or language of uncertainty. Examples include “I strive to find balance in my life,” “Values guide our decisions,” and “The environment is crucial for peace.”*

*A MetAware sentence refers to high-level abstraction, awareness, or consciousness of Concrete and Subtle phenomena, often questioning assumptions about reality. It focuses on complexity, philosophical considerations, and transcendent understanding. Examples include “Time is a construct shaping experience,” “Examining awareness reveals hidden biases,” and “Reality is shaped by our perceptions.” Identify the primary focus of the sentence and classify it accordingly.*

Is the sentence Individual or Collective?

*Classify the following sentence as (1) Individual, (2) Collective according to Terri O’Fallon’s classification protocol.*

*Individual Sentence (1): These sentences focus on single entity, personal experience, or individual actions.*

*Characteristics: Refers to a single person or object, often express with I or my, He or She of his or her and do not consider collective objects (family, group, community, collectivity, humanity, …). Describes personal experiences, qualities or actions of one individual. Example: I went for a walk, The cat is sleeping, She wrote an essay, the person listens.*

*Collective Sentence (2): A Collective sentence refers to objects that involve relationships, groups, processes, or systems encompassing multiple individual entities.*

*Characteristics: Presence of collective words: team, group, bus, train, family, community, people, others, collective, peers, world, they, humanity, organization, parties…. Example: The team won the game, Families gathered for the reunion. Processes and Systems: Discusses processes or systems that involve multiple individuals or entities. Example: The town’s economy is growing,” “Ecosystems rely on biodiversity. Cultural and Social Narratives: Describes value systems, cultural contexts, or societal narratives. Example: Cultural narratives shape our understanding of history, Religions provide moral frameworks. Relational and Perspective-taking: Involves early forms of relational understanding and empathy. Example: Imagining another’s pain can foster empathy, Helping others strengthens community bonds. Complex Systems and Interrelationships: Refers to complex systems, interrelationships of abstract concepts, or holistic world-systems. Example: Global warming affects ecosystems worldwide, Family dynamics influence individual behavior. For sentences that mix individual and collective subjects, identify where the main attention goes and classify the sentence accordingly.*

Is the sentence Active or Passive?

*Classify the following sentence as (1) Passive, (2) Active according to Terri O’Fallon’s framework for distinguishing agency: (1) Passive: The subject is receiving action, being influenced, or emphasizing receptivity and external causation. Language reflects a focus on being acted upon or experiencing without direct agency. (2) Active: The subject is the agent initiating action, exerting influence, or emphasizing doing and causation. Language reflects direct agency and intentional action.*

### Reaching deterministic results

Because the STAGES level is a unique result that combines the answers to the three questions, a variation in one of the answers leads to a difference and variations in two or three answers lead to important inaccuracy.

To reach a more deterministic result after the tuning of the prompt, central limit theorem (CLT) can be used considering that repeated sampling from a population will result in a distribution of the sample means. The median, being a measure of central tendency, can be considered a stable estimate of the performance of the model across runs (Kwak and Kim, 2017). Running several times the protocol and choosing the median value returned by the classification process is therefore an option supported by CLT.

To define the necessary number of runs required to reach a stable median value, a test has been performed on a training sample of 32 sentences using a regression-based method to determine the optimal number of runs required to achieve stable classification outcomes in a sentence classification task. The method involves analyzing changes in regression coefficients and constants across varying numbers of runs, aiming to identify the point at which additional runs no longer significantly influence the median classification outcomes. Multiple runs of a sentence classification script, ranging from 3 to 30 runs have been conducted. For each set of runs, the median classification outcome for three different questions (Q1, Q2, and Q3) and an aggregated STAGE level have been calculated. This process was repeated for subsets of runs starting from the minimum (three runs) up to 30 runs, increasing the starting point of each subset incrementally (series of 3 to 30 runs, series of 5 to 30 runs, …, series of 20 to 30 runs). For each subset of runs, we performed a linear regression analysis where the independent variable (*X*) was the number of runs and the dependent variable (*Y*) was the median classification outcome for each question and the STAGE level (Table 2).

**Table 2:** Result of the regression-based method.

	Question 1		Question 2		Question 3		STAGE level	
	*X*	Const.	*x*	Const.	*x*	Const.	*x*	Const.
3 runs	0.006	2.067	0.002	1.525	0.000	1.031	0.014	3.674
5 runs	0.013	2.050	0.003	1.522	0.000	1.031	0.028	3.638
10 runs	−0.003	2.109	0.006	1.516	0.000	1.031	0.000	3.750
15 runs	−0.016	2.135	0.016	1.500	0.000	1.031	−0.016	3.786
20 runs	0.000	2.094	0.063	1.438	0.000	1.031	0.063	3.641

The coefficients for Question 1 and Question 2 remain near 0, and for Question 3, a single run would be necessary. For the STAGE level, the optimal number of runs appears to be 10 as the curve completely flattened from 10 runs onward. This suggests that beyond 10 runs, additional runs do not significantly affect the median outcomes.

### Minimum number of stems required

As the purpose of the study was to explore the validity of a scoring process at scale on a larger population and group of people, the individual scoring of each sentence is not of major interest, an average value is preferred to position each respondent on the STAGES scale. With this approach, it may be not necessary to ask respondents to complete the 32 sentences of the sentence completion test (SCT).

**Figure 4:**
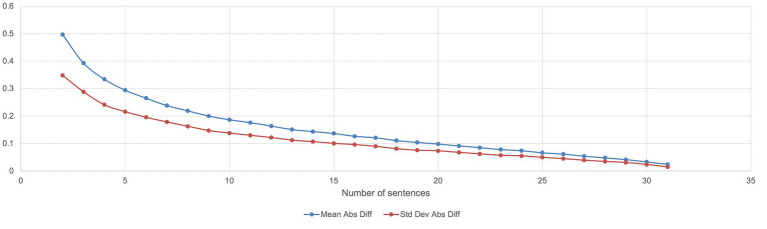
Evolution of the distance between the mean of the sample and the mean of a complete set.

Analysis has been performed to measure how the sample mean varies with different sample sizes ranging from 2 to 31 from a fixed dataset made of the 32 training sentences, to determine the influence of the sample size on the result comparing by measuring for each combination size, the mean absolute difference, and the standard deviation of absolute differences; 5,000 random combinations derived from the training dataset have been set as a basis for the analysis (Figure 4).

Considering the STAGES levels from 1 to 6.5 with a progression of 0.5, the optimal number of sentences to be considered for a valuation at scale would be 10 (standard deviation = 0.138; difference with the mean = 0.186 or 2.23% in scale value) considering that rounding of average value would lead to effective mean value. A minimum of five sentences may be accepted (standard deviation = 0.215; difference with the mean = 0.294 or 3.5% in scale value).

### Validation of the goodness of the protocol at scale with test sentences

A set of 58 sentences extracted from the study “Construction of a Scoring Manual for the Sentence Stem “A Good Boss--” For the Sentence Completion Test Integral (SCTi-MAP)” have been used (Miniard, 2009) together with sentences referenced in the study ego level in language (Lanning et al., 2018) (Table 3).

**Table 3:** Test sentences with MAP scores by experts (Miniard, 2009; Lanning et al., 2018) and STAGES scores computed by the protocol.

Id	Test sentences	MAP	STAGES scores
t-1	Women are lucky because in this day and age, we are finally moving up in the world and are respected	3.5	4
t-2	I feel sorry for people who do nothing with their lives	3.5	4
t-3	For me, development is something many people take too lightly these days	3.5	3
t-4	Women are lucky because they can usually think things out better than men who are sometimes irrational	3.5	4
t-5	Rules are there to protect us	2.5	2
t-6	For me, development is extremely hard but rewarding	3	1
t-7	The thing I like about myself is my hair	2	1
t-8	When I am criticized I become mad	2	1
t-9	For me, development is a complicated process but something well worth the effort	4	3
t-10	The thing I like about myself is my personality	2	1
t-11	The thing I like about myself is nothing	1.5	1.5
t-12	Education exists	1.5	2
t-13	I feel sorry myself	1.5	1.5
t-14	I feel sorry for very few people	1.5	2.5
t-15	Education should be valued over government	4.5	4
t-16	When people are helpless I do not know what emotion to feel or what to think about them – out of most peoples’ minds, sadly	4.5	4
t-17	Education is inefficient, but interesting socially – psychologists should be sub teachers at high school’s	4.5	4
t-18	The thing I like about myself is my concern to be honest with myself a claim that may itself be a delusion	5	3
t-19	The thing I like about myself is that I can let my child out; that I don t waste time judging the individuals I meet; and that I think independently and creatively	5	3
t-20	The thing I like about myself is I am stable and more independent that others my age	3.5	1.5
t-21	What gets me into trouble is boys	1.5	1
t-22	Women are lucky because we never have to be homophobic	4	4
t-23	Education is a long process	4	4
t-24	A man’s job is to make money	1.5	1
t-25	A man’s job is to work	1.5	1
t-26	A man’s job 50 years ago is anyone’s job today	4	4
t-27	My mother and I respect each other. She respects my decisions and independence and I respect her worry over me, she cannot help it!	4.5	4.5
t-28	My mother and I embrace each other’s differences and encourage each other	4.5	4.5
t-29	A good boss Is your friend	3	1
t-30	A good boss gets the job done and treat people fairly	4	2.5
t-31	A good boss is one who understands his employees, is patient and tolerant	4	4
t-32	A good boss is a person who both leads and treats his staff fairly	4	4
t-33	A good boss should be firm, gentle, considerate and interested in his staff	4	4
t-34	A good boss is one who is considerate and cares about the well-being of those who work for him	4	4
t-35	A good boss allows workers time to develop	4	4
t-36	A good boss provides opportunities for learning	4	4
t-37	A good boss is coaching people to become their best	4	4
t-38	A good boss is a judgment from a point of view (i.e., what is good?); at this moment I feel myself a “good boss” if I can be/act in the spirit of Lao Tsu, wherein a ‘good’ leader has such finely honed sensitivity, and is so invisible that he seems to do nothing special, yet accomplishes goals in a way that “People say ‘we did it!’”	5.5	5
t-39	A good boss Is a term that takes on new “bells and whistles” during this period of heightened technological and economic dominance; however, the primary concern for addressing the complex interplay of purposefulness and relatedness have remained similar since the time of the Greeks, Romans, Indians and Chinese.	5.5	6
t-40	A good boss is a boss God, that is, uses authority without presence of self but rather Self and the resulting Wisdom and Compassion and Flow enriches the field and otherwise becomes invisible as each person tends to translate experience in a healthier manner.	6	6
t-41	A good boss Is someone who knows how to balance authority and freedom, who is able to embody and/or give voice to the deeper mission of the organization, while seeing it in the context of society, and can do this in service of a deeper Reality.	6	4.5
t-42	A good boss is in the eye of the beholder, where the eye may be more than one “eye” (the subordinate, the peer, the boss him/herself, the superior, etc.) It remains relative until it is fixed in time and space by one or more than one.	6	6
t-43	When a child will not join in group activities, he may experience discomfort in interacting with the other children because of perceived differences leading to a non-homologation posture and/or because he feels non-assimilated nor recognized by the group.	3.5	3
t-44	When I get angry, it provides me with good input to do shadow work on myself considering the triggering party as the mirror of what’s going on inside. It’s not about the others but it’s about me. It’s not about changing the others but about making sense of the experience	4	3.5
t-45	The thing I like about myself is the curiosity and ability to integrate the constituents of the body of knowledge and wisdom. Curiosity is stimulated by the community around me that help me opening windows and doors I did not even suspect.	4	3.5
t-46	If my mother grow me as she did, it’s because of her best intention and knowledge. It’s up to me to welcome and embrace her education, keep what I like and transform what does not perfectly fit.	4	3.5
t-47	When people are helpless, it’s the right moment for them to letting go. We may consider helplessness as a relative concept connected to the feeling of being a victim or not and letting go being a tool to get out of the victim posture.	4	4
t-48	Education is the vehicle to support development, it provides the necessary external consciousness to individuals and groups to integrate new knowledge, postures, feelings, habits, abilities and wisdom.	4	4.5
t-49	At my worst, when life is not enough to complete my own development, I will have to come back for another round.	3.5	3.5
t-50	I feel sorry for all the people on Earth who experience material difficulties in life and have to fight for their fundamental rights and I feel sorry in the meantime for all the people on Earth who pursue materialistic goals in a never enough way of thinking and behaving.	4	4
t-51	Raising a family is a majestic experience that provides parents with the opportunity to support the development of cells by creating the conditions for optimal development prior to releasing them into the world, a bit like being in charge of a cultured broth.	4	4.5
t-52	Rules are sometimes necessary when the collectivity experiences difficulties in establishing the balance between personal and collective interests. They should be adapted to the collective level of consciousness of the group and just be good enough.	4	4
t-53	Being with other people creates the opportunity for me to explore other ideas, discover new pointers to peculiar knowledge. Should the group be felt as a safe place to be, it provides the pleasant sensations of being in a safe harbor to regenerate and relax.	4	4
t-54	If I had more money I would be able to increase the impact of the research work I contribute to. I would also be able to help others reaching a better place to live. This would also bring me to a safer place for my old time.	4	3.5
t-55	People who step out of line at work may want to challenge the status quo, to suggest new way of getting the things done considering that traditions represent a hindrance to development. They may also do that because of moral issues such as inequality of treatment or perceived forced consent; in this case they feel the urge to act on behalf of others.	4	4.5
t-56	If I cannot get what I want, it’s an amazing invitation to sublimate my impulses and desires. Shouldn’t I be able to welcome it, I’m looking for another way forward.	4	3
t-57	I’d love to see humanity resolving the current challenges embracing a humanistic posture and integral way of thinking.	4	6.5
t-58	A healthy organization is always working for the interest of all the involved parties, develops an ecological vision, builds strategy around people leveraging on a set of humanistic and positive values, and integrates clients and suppliers. The healthy organization uses capital as a mean, not an end.	4	4.5

To verify the agreement between scoring of MAP experts and STAGES protocol at scale, Cohen’s kappa has been measured (Tables 4, 5).

**Table 4:** MAP × STAGES at scale cross-tabulation between scoring of MAP experts and STAGES process at scale.

		STAGES scores												
		1	1.5	2	2.5	3	3.5	4	4.5	5	5.5	6	6.5	Total
MAP	1	0	0	0	0	0	0	0	0	0	0	0	0	0
	1.5	3	2	1	1	0	0	0	0	0	0	0	0	7
	2	3	0	0	0	0	0	0	0	0	0	0	0	3
	2.5	0	0	1	0	0	0	0	0	0	0	0	0	1
	3	2	0	0	0	0	0	0	0	0	0	0	0	2
	3.5	0	1	0	0	2	1	3	0	0	0	0	0	7
	4	0	0	0	1	2	4	14	4	0	0	0	1	26
	4.5	0	0	0	0	0	0	3	2	0	0	0	0	5
	5	0	0	0	0	2	0	0	0	0	0	0	0	2
	5.5	0	0	0	0	0	0	0	0	1	0	1	0	2
	6	0	0	0	0	0	0	0	1	0	0	2	0	3
	6.5	0	0	0	0	0	0	0	0	0	0	0	0	0
	Total	8	3	2	2	6	5	20	7	1	0	3	1	58

**Table 5:** Agreement (Kappa) between MAP and STAGES scores at scale.

Symmetric measures				
	Value	Standard error	95% CI	
Agreement (weighted kappa with quadratic weights)	0.77987	0.05491	0.67225	0.88749
N of valid cases	58			

The inter-rater agreement is measured using quadratic weights kappa measure considering the ordinal nature of the scale treating larger disagreements with higher weights than smaller ones (Vanbelle, 2016).

The weighted Cohen’s kappa for the inter-rater agreement is 0.77987, indicating substantial agreement between the raters. The standard error associated with the kappa statistic was 0.05491, and the 95% confidence interval ranged from 0.67225 to 0.88749. This confidence interval does not include zero, demonstrating that the observed agreement is significantly greater than what would be expected by chance. The results suggest a strong level of consistency in the ratings provided by the raters (Cohen, 1960).

To assess the validity of consolidated scores to work on individual averages, 5,000 combinations of 10 sentences have been established using the test sentence dataset. The average values of those 5,000 combinations have been rounded to 0.5 values to stick to the STAGES scale. The agreement between average values from experts and from the protocol has been measured based on Cohen’s weighted kappa (Table 6).

**Table 6:** Agreement (Kappa) between MAP and STAGES scores on average values for 5,000 random combinations of 10 sentences.

Symmetric measures				
	Value	Standard error	95% CI	
Agreement (weighted kappa with quadratic weights)	0.7052	0.0100	0.68560	0.72490
*N* of valid cases	5,000			

A substantial agreement on average scores is observed (weighted Cohen’s kappa for the inter-rater agreement is 0.7052, standard error of 0.01, and 95% confidence interval ranging from 0.68560 to 0.72490) illustrating a substantial goodness of aggregated data, confirming the opportunity to work on consolidated scores.

## Discussion

The obtained weighted kappa statistic of 0.77987 highlights several critical implications for the reliability of the manual and automated classifications. The kappa value falls within the range described as “substantial agreement,” indicating some level of concordance between the two methods (Cohen, 1960). Although this signifies that there is a good degree of consistency, the differences observed suggest that the automated process might not be entirely reliable in replicating expert assessments for single sentences.

Several factors could contribute to this substantial agreement. First, classification tasks often involve complex patterns and subtleties that automated processes may struggle to capture compared to human experts. This is particularly likely in scenarios where contextual understanding or nuanced judgment is required. The automated process, possibly reliant on preset algorithms or machine learning models, might miss out on such intricacies, leading to discrepancies in classification.

Moreover, the standard error of 0.05491 implies that the kappa value has a narrow margin of variability, further emphasizing that our point estimate of 0.77987 is reliable. The results of the study underline the potential of the classification process at scale. Alongside the constant evolution of LLMs, the protocol may be further enhanced. Strategies might include refining the algorithm, fine-tuning the selected LLMS or combining expert reviews to periodically recalibrate the system. Enhancing the complexity and adaptability of the classification model could also aim to reduce the observed discrepancies.

With a good level of agreement observed for single-sentence scoring, our analysis indicates that the automated process exhibits low variability in aggregated values as well. This is particularly advantageous when calculating ego development scores for larger entities such as individuals, groups, corporate units, or entire corporations. At these aggregated levels, the minor discrepancies in individual sentence classifications tend to balance out, resulting in more stable and reliable overall scores. The robustness of the automated process at scale ensures that it is effective for high-level evaluations and decision-making, with significant utility in organizational psychology, corporate analytics, and developmental assessments. This boosts the goodness of the process for large-scale applications where aggregated data are paramount, thereby providing valuable insights that are consistent and actionable over broader contexts.

The methodology employed in this study, which combines the selection of the large language model, the prompt engineering of training data, the definition of minimal content to obtain significant results, and finally Cohen’s kappa statistic with a paired analysis of expert and automated classifications from a test dataset, provides a framework for evaluating the reliability of automated processes. By using Cohen’s weighted kappa, we account for chance agreement, making our assessment more rigorous than simple percent agreement measures. Additionally, the significance testing adds a quantitative element that underscores the credibility of the findings. This structured approach offers a comprehensive means of benchmarking automated systems against human experts, ensuring that any identified gaps are not attributed to random variance. This methodology is particularly effective for large-scale data evaluations where aggregated insights are crucial, as it allows for systematic identification and correction of discrepancies, thereby promoting continuous improvement in automated processes.

### Limitations and future research

In this case study, the test dataset size of 58 valid cases may limit the generalizability of the results but serve the purpose of the research work. Future research may consider larger sample sizes to enhance the robustness of the findings.

Future research directions include applying advanced machine learning techniques, incorporating continuous feedback loops from experts, and expanding the dataset to include more diverse cases. Emphasizing the interpretability and transparency of the automated models could also help in identifying specific areas where the automated process diverges from expert opinions, thereby facilitating targeted improvements.

By addressing these limitations and expanding our methodological toolkit, future studies can work toward automated classification systems that closely emulate expert assessments, leading to more accurate and reliable outcomes in automated data processing tasks.

## Conclusion

In conclusion, this study explored the agreement between expert and automated classifications using Cohen’s weighted kappa statistic. The kappa value of 0.779 indicates a good level of agreement on individual scores, and a kappa value of 0.705 on consolidated averages. These findings highlight the potential of the automated process even if further refinements to achieve higher accuracy in line with expert assessments may be desirable to use the protocol on single-sentence analysis.

The process demonstrates robustness in aggregated data contexts. The low variability observed when deriving aggregated values, such as ego development scores for individuals, groups, corporate units, or entire corporations, supports the effectiveness of the automated process for large-scale applications. This suggests substantial utility in fields such as organizational psychology and corporate analytics, where aggregated insights often drive decision-making and strategy formulation.

Moving forward, improving the automated system involves refining the underlying algorithms, incorporating more comprehensive training datasets, and integrating periodic expert feedback to recalibrate the system. The contextual adaptability of the automated process must also be enhanced to capture the nuanced judgments that experts readily identify.

Future research may focus on enhancing the scoring precision on individual sentences, applying advanced machine learning techniques for better precision, and ensuring transparency in the automated methods. Addressing these avenues will pave the way for more accurate, reliable, and scalable automated classification systems that can closely emulate expert evaluations.

By leveraging these improvements, we anticipate that the automated classification systems will achieve a level of accuracy and reliability that meets the high standards required for extensive and practical applications. This advancement will ultimately lead to more consistent, actionable insights derived from aggregated data, further enhancing the value and applicability of automated processes in diverse fields.

Finally, the methodology used in this case study appears to be useful to support other evaluations at scale that leverage on large language model using other classifications’ maps.

## Data Availability

The raw data supporting the conclusions of this article will be made available by the authors, without undue reservation.
